# Natural History, &c.

**Published:** 1840-01-01

**Authors:** 


### NATURAL HISTORY, &c,

I. A HISTORY OF BRITISH REPTILES. By *Thomas Bell*, F.R.S. F.L.S. Professor of Zoology in King's College, London. Illustrated by a wood-cut of each species, with some of the varieties, and numerous vignettes. Van Voorst. Part III. completing the work.

This interesting account of British Reptiles is now concluded. It makes one of that series of works, published by the enterprising bookseller to the Zoological Society, Mr. Van Voorst. We have often spoken in deservedly favourable terms of preceding Parts. We can speak equally favourably of this. In conjunction with it we may notice the following Parts of other works of the same complexion. The later Parts have not yet reached us. We shall take care to make mention of them to our readers when they do.

II. A HISTORY OF BRITISH BIRDS. By *William Yarrell*, F.L.S. F.Z.S. Parts XIII. and XIV.

This maintains its character—no slight encomium. The chough and crow, the raven, and the rook, *caw* in the pages before us—and the jackdaw, the jay, and the woodpeckers are there too.

III. A GENERAL OUTLINE OF THE ANIMAL KINGDOM AND MANUAL OF COMPARATIVE ANATOMY. By *Thomas Rymer Jones*, F.Z.S. Professor of Comparative Anatomy in King's College, London. Part VII.

This very useful as well as agreeable work holds on its way. The execution is equally creditable to author and to publisher. The present Part is occupied with insecta, arachnida, and crustacea.

IV. SUPPLEMENT TO THE HISTORY OF BRITISH FISHES. By *William Yarrell*. Illustrated with wood-cuts. In two Parts.

It is indeed gratifying to see this interest in Natural History—we trust that medical men will be foremost in evincing it. From the nature of their studies they are adapted to relish and to advance it. We think this series of publications well calculated to foster such a taste.

